# The Serum Metabolite Response to Diet Intervention with Probiotic Acidified Milk in Irritable Bowel Syndrome Patients Is Indistinguishable from that of Non-Probiotic Acidified Milk by ^1^H NMR-Based Metabonomic Analysis 

**DOI:** 10.3390/nu2111141

**Published:** 2010-11-23

**Authors:** Simon M. M. Pedersen, Niels Chr. Nielsen, Henrik J. Andersen, Johan Olsson, Magnus Simrén, Lena Öhman, Ulla Svensson, Anders Malmendal, Hanne C. Bertram

**Affiliations:** 1 Department of Food Science, Faculty of Agricultural Sciences, Aarhus University, Blichers Allé 20, P.O. Box 50, DK-8830 Tjele, Denmark; Email: simonmm.pedersen@gmail.com (S.M.M.P.); hannec.bertram@agrsci.dk (H.C.B.); 2 Center for Insoluble Protein Structures, Interdisciplinary Nanoscience Center (iNANO) and Department of Chemistry, University of Aarhus, Langelandsgade 140, DK-8000 Aarhus C, Denmark; Email: ncn@chem.au.dk (N.C.N.); 3 Arla Foods amba, P.O. Box 2400, DK-8260 Viby J, Denmark; Email: henrik.jorgen.andersen@arla.dk (H.J.A.); ulla.svensson@arlafoods.com (U.S.); 4 KPL Good Food Practice AB, Dag Hammarskjölds vag 10 B 3tr, 751 83 Uppsala, Sweden; Email: johan.olsson@good-food-practice.com (J.O.); 5 Department of Internal Medicine, Bruna Stråket 11, Sahlgrenska University Hospital, 413 45 Gothenburg, Sweden; Email: magnus.simren@medicine.gu.se (M.S.); lena.ohman@microbio.gu.se (L.Ö.)

**Keywords:** metabonomics, ^1^H-NMR spectroscopy, Irritable Bowel Syndrome, acidified milk products, multivariate data analysis, lactate, probiotics

## Abstract

The effects of a probiotic acidified milk product on the blood serum metabolite profile of patients suffering from Irritable Bowel Syndrome (IBS) compared to a non-probiotic acidified milk product was investigated using ^1^H NMR metabonomics. For eight weeks, IBS patients consumed 0.4 L per day of a probiotic fermented milk product or non-probiotic acidified milk. Both diets resulted in elevated levels of blood serum L-lactate and 3-hydroxybutyrate. Our results showed identical effects of acidified milk consumption independent of probiotic addition. A similar result was previously obtained in a questionnaire-based evaluation of symptom relief. A specific probiotic effect is thus absent both in the patient subjective symptom evaluations and at the blood serum metabolite level. However, there was no correspondence between symptom relief and metabolite response on the patient level.

## 1. Introduction

Irritable Bowel Syndrome (IBS) is a very common gastrointestinal disorder associated with abdominal pain connected with defecation [1]. Abnormal defecation such as diarrhea and constipation along with distension are major symptoms, which do not coincide with demonstrable abnormalities [2]. Most patients manage to keep discomfort at a minimum by regular exercise, low-fat, fiber-rich diets, and medication. However, some patients suffer from severe symptoms that influence the quality of their everyday life such as travel over short distances and social life [3]. 

During the past decade, several studies [4,5,6,7,8,9] have shown that consumption of probiotics has positive effects on gastrointestinal inflammation, thereby conferring benefits to people suffering from IBS. These effects have been identified using symptom questionnaires in combination with either measurements of colonic transit [4] or collection of fecal and blood samples [7,8,9,10]. Intake of probiotics has also shown to delay the first onset of pouchitis and to improve the general quality of life for patients undergoing ileal pouch-anal anastomosis [5,6]. High placebo response is reported based on questionnaires [10,11,12,13], which raises the question of the placebo product or the probiotic delivering matrix having an influence in itself. 

Over the past few years, metabonomics based on proton NMR (^1^H NMR) coupled with multivariate data analysis has proven to be an extremely powerful method for determination of variations in metabolite concentrations within data collected from large numbers of samples [14,15,16]. With a minimum of sample preparation, high reproducibility [17], and short experimental time, it can be considered a superior approach providing dietary intervention studies with a tool for detection of metabolite changes associated with the experimental diet. 

As a secondary investigation within a project where blood serum samples from IBS patients enrolled in a large controlled double-blind diet intervention study [10] were collected, the aim of the present study was to investigate the influence of the consumption of acidified milk products with probiotics on the blood serum metabolite composition. Using a metabonomic approach based on ^1^H NMR spectroscopy coupled with multivariate statistical analysis, we analyzed the change in the blood serum metabolite profile in IBS patients caused by intake of either a probiotic fermented milk product or a non-probiotic acidified milk product. 

## 2. Experimental Section

### 2.1. Study participants and design

An initial group of volunteer IBS patients was enrolled in the study based on the Rome II criteria [18]. Patients were then followed for two weeks to establish whether they truly matched the Rome II criteria and to study symptom severity. Symptoms were scored according to the IBS severity scoring system (SSI) [19]. Study inclusion required that the patients had to score VAS (Visual Analogue Scale) > 40 on at least one of five questions of the IBS SSI questionnaires and to have a lack of adequate relief of the IBS symptoms the week before inclusion. Patients not fulfilling these criteria were excluded from the study, leaving a final group of 74 subjects. Subjects were randomized for one of two treatments. During the intervention period, seven subjects dropped out for reasons unrelated to the study. For different reasons, blood serum samples were not acquired from six subjects, leaving a total of 61 subjects in the final group completing the study. This total patient group consisted of 45 females and 16 males aging from 18 to 79 years with a mean of 42.5 years. Data on age, BMI, gender, time with IBS and type of IBS for the patients per group was collected (see additional file: Supplementary Table 1). All patients gave their informed consent. The study was performed in accordance with the Declaration of Helsinki II and approved by the Ethics Committee of the University of Gothenburg. The trial was registered at ClinicalTrial.gov with the registration number: NCT01127828. 

### 2.2. Dietary treatment and sample collection

Two different dietary treatments consisting of a fermented dairy product containing probiotics and a chemically acidified milk product, respectively, were used. The probiotic acidified milk product (Cultura^©^, Arla Foods amba, Denmark) was produced by fermentation of homogenized, high-pasteurized, low-fat (1.5%) milk by the use of two fermentation starter cultures, *Lactobacillus delbruckeii* ssp. *bulgaricus* and *Streptococcus thermophilus* and the inclusion of three probiotic strains, *Lactobacillus paracasei* F19, *Lactobacillus acidophilus* LA-5 and *Bifidobacterium lactis* BB-12, at concentrations of 5 × 10^7^ CFU/mL (see additional file: Supplementary Table 2). The chemically acidified product was produced by acidification of homogenized, high-pasteurized, low-fat (1.5%) milk by addition of D-(+)-gluconic acid δ-lactone (≥99.0%) (GDL) (Sigma-Aldrich, Seelze, Germany; see additional file: Supplementary Table 2). Addition of GDL mimics the slow pH reduction created during fermentation and produces a product comparable to the probiotic product in taste, texture and color. Both products had a pH value of 4.5. Before entering the study, patients had a wash-out period where they did not consume any acidified milk products for a 2-week period. After this period, the two patient groups were instructed to consume 0.4 L per day, divided in two takings of 0.2 L, of either the GDL-acidified milk product (*n* = 31) or the probiotic acidified milk product (*n* = 30) over an 8-week period. In this report, the two products will be referred to as the GDL milk product and the probiotic milk product. During the intervention period, patients were instructed not to consume any other fermented dairy products. Blood samples were collected, the body weight measured, and intake of energy, protein, fat, carbohydrates, calcium, and fiber in a 3-day period were recorded (Table 1) for each subject one day before the trial started (baseline) and after the last day of the trial period (post treatment). 

**Table 1:** Summary of 3-day energy intake record by IBS patients before sample collection *^a^* and IBS patients’ body weight measurements. Patient data from samples included in the ^1^H-NMR study.

Nutrient	GDL milk product *^b^*	Probiotic milk product *^c^*
Energy, *kJ/(kg body wt) ^d^*		
Baseline	31.07 ± 1.57	28.25 ± 1.50
Post treatment	27.78 ± 1.86 *	24.24 ± 1.25 *
Protein, *g/(kg body wt)*		
Baseline	1.22 ± 0.08	1.12 ± 0.05
Post treatment	1.04 ± 0.07 *	0.95 ± 0.04 *
Fat, *g/(kg body wt)*		
Baseline	1.21 ± 0.09	1.04 ± 0.07
Post treatment	0.98 ± 0.09 *	0.89 ± 0.05 *
Carbohydrates, *g/(kg body wt)*		
Baseline	3.62 ± 0.18	3.34 ± 0.19
Post treatment	3.31 ± 0.23 *	2.89 ± 0.19 *
Calcium, *mg/(kg body wt)*		
Baseline	16.06 ± 1.19	13.71 ± 1.14
Post treatment	12.68 ± 1.24 *	10.07 ± 1.00 *
Fiber, *g/(kg body wt)*		
Baseline	0.31 ± 0.02	0.26 ± 0.01
Post treatment	0.26 ± 0.03 *	0.21 ± 0.01 *
Weight, *kg*		
Baseline	73.13 ± 2.58	72.17 ± 2.74
Post treatment	74.11 ± 2.75 *	72.51 ± 2.74

*^a^* Nutritional data are given as mean ± SEM of summed 3-day nutritional intake. Baseline data was obtained 1 day before initiation of the intervention period and post treatment data was collected on the last day of the intervention period. Body weight measurements are given as mean ± SEM. Post treatment means marked with * differ significantly from baseline (*P* ≤ 0.05);

*^b^ n* = 28. One patient failed to record nutritional data before collection of the baseline sample and was therefore left out of the analysis;

*^c^* *n* = 29. One patient failed to record nutritional data before collection of the post treatment sample and was therefore left out of the analysis;

*^d^* Patient energy intake at baseline and post treatment differed not significantly (*P* > 0.05) between GDL milk product and probiotic milk product patient groups.

The patients fasted overnight and then consumed a fiber-rich non-dairy meal (Content: 2258 kJ; 36% fat, 15% proteins, 49% carbohydrates; 9.2 g fiber) 1 h before collection of blood. The meal was given to the patients to study possible symptom relief induced by the consumption of the two different acidified milk products. Blood serum was prepared by collecting 5 mL blood from the antecubital vein in silicon-treated Vacutainer^®^ tubes. The blood was left to clot for 30 min followed by 20 min centrifugation at 1600 × *g*. The serum fraction was collected and stored in aliquots at −70 °C. 

### 2.3. D- and L-lactate measurements

Five GDL milk group and six probiotic milk group baseline samples and their corresponding post treatment samples were randomly selected for lactate isomer analysis. Proteins larger than 10 kDa were removed by filtrating 250 μL of each sample using a Microcon 10-YM Centrifugal Filter Unit (Millipore, Billerica, USA) according to the manufacturer’s protocol. D- and L-lactate concentrations were measured using a D-lactic acid/L-lactic acid kit (Boeringer Ingelheim GmbH, Ingelheim am Rein, Germany) according to the manufacturer’s protocol.

### 2.4. Sample preparation and ^1^H NMR analysis

Serum samples were prepared by adding D_2_O (serving as a spectrometer frequency lock) into 200 µL of serum to a total volume of 600 µL. ^1^H NMR measurements were performed on a Bruker Avance II 700 spectrometer (Bruker Biospin, Rheinstetten, Germany), with a standard ^1^H detection 5 mm HCN triple resonance probe, at 25 °C and with a ^1^H frequency of 700.09 MHz (16.4 T). ^1^H spectra were acquired using a single-90°-pulse experiment with a Carr–Purcell–Meiboom–Gill (CPMG) delay. The CPMG delay removes broad signals from high-molecular-weight molecules that would otherwise complicate interpretation of the spectra. Total CPMG delay was 40 ms and the spin‑echo delay 200 µs. Water was suppressed by presaturation of the water peak during the relaxation delay of 1.5 s. A total of 128 transients of 16 K data points spanning a spectral width of 24 ppm were collected. Experimental time for collection of one spectrum was 5 min. For assignment purposes, a two-dimensional (2D) ^1^H-^1^H TOtal Correlation SpectroscopY (TOCSY) spectrum [20] with 80 ms mixing was acquired on a representative sample.

### 2.5. Data processing and statistical analysis

Processing of ^1^H NMR spectra was performed using the *iNMR* software (Nucleomatica, Molfetta, Italy). An exponential line-broadening of 0.5 Hz was applied to the free induction decay (FID) before Fourier transformation. All acquired spectra were referenced to the CH_3_ chemical shift of alanine at 1.466 ppm. Data reduction of the ^1^H NMR spectra was performed by dividing the spectra into 0.01 ppm regions, so-called bins. Each bin was then integrated to obtain the total signal intensity. The region from 10.00–0.00 ppm, except for the region comprising the water signal (5.11–4.66 ppm), was used for analysis. Normalization to total intensity of the spectrum was performed before further data analysis. Cross-validated principal component analysis (PCA) was performed on mean-centered data to identify discrete patterns. PCA reduces the dimensionality of a data set by finding a new set of variables, smaller than the original set of variables, and retains most of the sample’s information. The new variables, called principal components, are uncorrelated, and are ordered by the fraction of the total information they explain. Each principal component is described by a loading vector, like an NMR spectrum with positive and negative peaks, and scores that describe how much the loading vector contributes to the spectrum of each sample. The scores can be used to identify similarities and differences between samples. Patterns identified by PCA were subsequently analyzed by cross‑validated orthogonal-projection to latent structures-discriminant analysis (OPLS-DA) [21]. In the cross validated OPLS-DA algorithm, the variation in the ^1^H NMR data is decomposed into three parts: the variation of the data correlated to the discriminant class, the specific systemic variation not correlating to the discriminant matrix and the residual variation. Thereby a model with minimum predictive components defined by the number of degrees of freedom between group variances was produced. Cross validation was performed by removing every seventh sample and the new data set was used to predict the excluded samples. The process was replicated until each sample had been removed at least once. The optimal number of components is based on the cross validation test and the value *Q^2^*(cum) describes the predictability of the model. When performing OPLS-DA on the ^1^H NMR spectral data, unit variance scaling was applied to allow signals of lower intensity to exert the same weight on the model as higher-intensity signals. To facilitate interpretation of the OPLS-DA covariances (loadings), back-scaling transformation and presentation as a limit discriminant weight plot were applied. Back-scaling was performed by calculation of the covariances between each bin and the predictive component scores.

The significance of each bin, and thereby the individual metabolites, was elucidated by calculation of the correlation coefficient of each bin and the corresponding predictive component score. Limit discriminant weight plots were then produced and these plots present covariances of variables where variables with a correlation coefficient squared, *R*^2^, to the class discrimination higher than 0.60 are highlighted. Multivariate data analysis was performed using the Simca-P 11.5 software (Umetrics, Umeå, Sweden). 

Data on record of 3-day dietary intake, body weight, and lactate measurements were analyzed statistically by paired *t* tests. One-way ANOVA was used for analyzing PCA scores for differences between baseline and post treatment samples. An unpaired *t* test was performed to analyze age and BMI differences between the two treatment groups. All tests were performed using the 0.05 significance level.

## 3. Results

**Figure 1:**
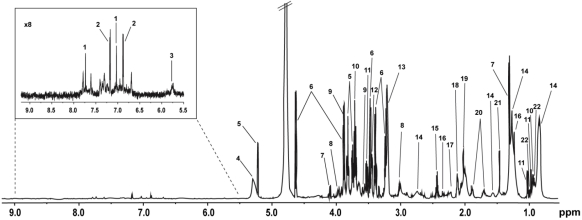
^1^H NMR CPMG spectrum of human blood serum from a patient suffering from IBS. The key is given in Table 2.

Human blood serum spectra were assigned by comparison of chemical shifts in a representative spectrum (Figure 1) to established libraries reported in the literature [22,23,24,25], the Human Metabolome Data Base (HMDB) [26], and confirmed by cross-peak assignment in the 2D TOCSY spectrum. The ^1^H NMR spectra contained 22 assignable metabolites including amino acids, lipids, organic acids, glycoproteins, and glucose (Table 2). Visual inspection of all spectra revealed no general, obvious differences in metabolite composition between samples. However, one GDL baseline sample displayed an atypical spectrum with broad and strong peaks and extra peaks assigned to lipid metabolites. Data regarding nutritional intake, disease or medication could not account for this atypical appearance. This sample and the corresponding post treatment sample were therefore discarded from further analysis. 

**Figure 2:**
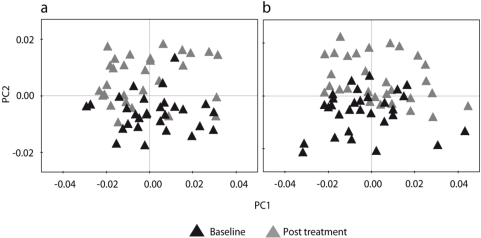
PCA score plot of baseline and post treatment samples (a) GDL acidified milk patient group baseline and post treatment samples (*n* = 58). *R^2^X*(1) = 0.516, *R^2^X*(2) = 0.178 and *Q^2^*(cum) = 0.941; (b) Probiotic milk patient group baseline and post treatment samples (*n* = 60). *R^2^X*(1) = 0.556, *R^2^X*(2) = 0.200 and *Q^2^*(cum) = 0.928. Post treatment different from baseline, *P* ≤ 0.05. *R^2^X* describes how much of the variance that is explained by the component. *Q^2^*(cum) represents the predictability of the total model and is related to the statistical validity of the model.

With the aim of identifying more discrete patterns and changes, multivariate data analysis was performed. PCA revealed another GDL baseline sample outlier, due to very large and broad lipid signals. This sample, together with its complementary post treatment sample, was discarded from further analysis to avoid distortion of the multivariate data models. Thus, the resulting dataset contained 58 samples from 29 patients in the GDL milk group and 60 samples from 30 patients in the probiotic milk group. In order to investigate the variation in the baseline and post treatment samples, PCA was performed on the GDL milk and the probiotic milk baseline and post treatment groups, respectively (see additional file: Supplementary Figure 1 and Figure 2). The PCA scores were tested for groupings according to gender, age and BMI (data not shown). No patterns were detected in either of the groups in any of the performed tests. We also tested for treatment response. Although this subgroup had shown symptom relief in both treatment groups [10], no distinction could be made between responders and non-responders based on blood serum metabolite composition (data not shown). The tests were repeated using OPLS and OPLS-DA without detecting any correlations. Accordingly, the results demonstrated that each group could be considered as one, containing no sub groupings. 

**Table 2:** Assignments of observed ^1^H-NMR peaks in blood serum of a patient suffering from IBS.

Key	Metabolite	δ _1H_ (multiplicity)	Moieties
1	Histidine	7.74 (s), 7.03 (s)	CH, CH
2	Tyrosine	7.17 (m), 6.87 (m)	CH, CH
3	Urea	5.76 (s)	NH_2_
4	Unsaturated lipids in serum	5.28 (m)	-CH=CH-
5	α-Glucose	5.22 (d), 3.82 (m), 3.71 (t), 3.52 (d)	C-H1, C-H6, C-H3, C-H2
6	β-Glucose	4.63 (d), 3.89 (d), 3.47 (d), 3.40 (t), 3.23 (d)	C-H1, C-H6, C-H5, C-H4, C-H2
7	Lactate	4.10 (q), 1.32 (d)	αCH, βCH_3_
8	Creatine	3.92 (s), 3.04 (s)	CH_2_, NCH_3_
9	Glycerol	3.88 (m), 3.65 (d)	CH, CH_2_
10	Leucine	3.70 (m), 0.96 (d)	αCH, γCH_3_
11	Valine	3.60 (d), 1.03 (d), 0.97 (d)	αCH, γCH_3_, δCH_3_
12	Proline	3.44 (t)	δCH_2_
13	Choline	3.21 (s)	N(CH_3_)_3_
14	Lipid	2.73 (m), 1.56 (m), 1.25 (m), 0.84 (m)	C=CCH_2_C=C, CH_2_CH_2_CO, (CH_2_)n, CH_3_(CH_2_)n
15	Glutamine	2.43 (m)	γCH_2_
16	3-hydroxybutyrate	2.34 (m), 1.20 (d)	αCH_2_, βCH_3_
17	Acetoacetate	2.21 (s)	CH_3_
18	Glutamate	2.10 (m)	βCH_2_
19	*N*-acetyl glycoproteins	2.03 (s)	CH_3_
20	Lysine	1.88 (m), 1.72 (m)	βCH_2_, δCH_2_
21	Alanine	1.46 (d)	βCH_3_
22	Isoleucine	0.99 (t)	βCH_3_

After establishing that baseline and post treatment groups had no sub-groupings, PCA was carried out on the sample sets containing both baseline and post treatment samples (Figure 2). For both the GDL acidified milk group and the probiotic milk group principal component 2 showed a significant separation of baseline and post treatment samples using one-way ANOVA. Both the GDL and the probiotic treatment groups showed a similar discrimination of baseline and post treatment samples. To identify the difference between baseline and post treatment samples, cross validated OPLS-DA was conducted in parallel on the GDL acidified and probiotic milk group using baseline and post treatment as the discriminant matrix (see additional file: Supplementary Figure 3).

**Figure 3:**
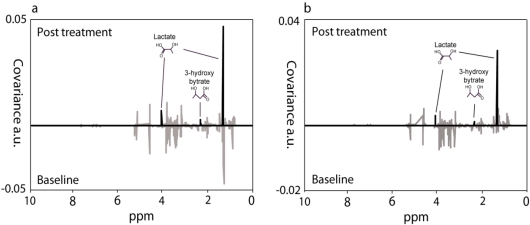
Metabolite identification based on limit discriminant weight plots. Limit discriminant weight plots of back-scaled OPLS-DA data from GDL milk and probiotic milk treated patient groups with variable covariances presented with different *R^2^* cut-off values. (a) GDL acidified milk group; (b) Probiotic milk group. Peak at δ 1.32 ppm represents the β_CH3_ of lactate and the peak at δ 4.10 ppm represents the α_CH_ of lactate. The peak at δ 2.34 ppm represents the α_CH2_ proton of 3-hydroxybutyrate. Grey spectra show all covariances and black spectra have a cut-off value *R*^2^ > 0.6.

High *R^2^X* (GDL *R*^2^*X*(cum) = 0.821 and probiotic *R*^2^*X*(cum) = 0.833) and *Q^2^* values (GDL *Q*^2^(cum) = 0.841 and probiotic *Q*^2^(cum) = 0.848) using one predictive and four orthogonal components in both analyses reveals statistically robust and highly predictable models. A clear discrimination between the baseline and post treatment groups indicated that a large majority of the patients had consumed their prescribed experimental diet satisfactorily. In order to identify metabolites responsible for the separation between baseline and post treatment samples, OPLS-DA loadings were evaluated by calculating the correlation and the covariance for each variable with the OPLS-DA predictive scores. The resulting plots display the variable covariances (Figure 3). 

**Figure 4:**
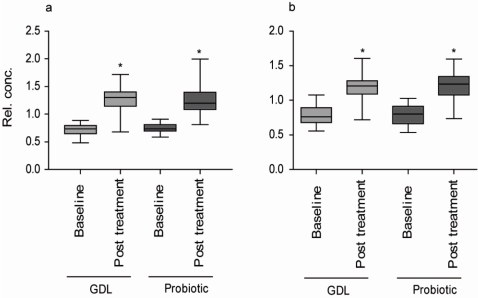
Relative concentration of lactate and 3-hydroxybutyrate based on signal intensity. (a) Lactate; (b) 3-hydroxybutyrate. Boxes represent 25th to 75th percentile of data and whiskers represent data spread. Asterisk (*) indicate that post treatment value is significantly different from baseline, *P* ≤ 0.05.

The plots show variables that are significantly correlated (*R^2^* > 0.6, black spectra) with the separation of baseline and post treatment samples. The results for the GDL milk group and for the probiotic milk group are virtually identical. In both plots, three peaks showed high correlations with the discrimination; the α_CH_ of lactate at 4.10 ppm, the α_CH2_ proton of 3-hydroxybutyrate at 2.34 ppm and the β_CH3_ of lactate at 1.32 ppm. No other metabolites had *R^2^* values higher than 0.45, emphasizing that lactate and 3-hydroxybutyrate were particularly important for the effect induced by the two treatment types. Both ^1^H NMR proton signals from lactate were present. Only the α_CH2_ from 3‑hydroxybutyrate had a high correlation in the discriminant weight plot due to overlap of the β_CH3_ with large lipid signals around 1.20 ppm. Positive covariances show that the level of lactate and 3‑hydroxybutyrate was higher in the post treatment samples than in the baseline samples. The relative concentrations of lactate and 3-hydroxybutyrate as measured by the intensity of the signals in the NMR spectra are shown in Figure 4. To confirm that lactate was responsible for the separation between baseline and post treatment samples, the relevant variables were extracted and used for PCA. Both in the GLD milk group and the probiotic milk group clear separation of baseline and post treatment samples were observed in PCA score plots when only using variables for lactate (data not shown).

**Figure 5:**
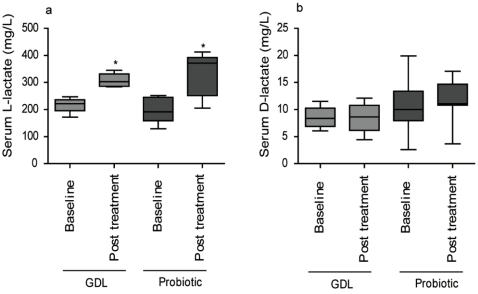
D- and L-lactate measurements. GDL acidified milk patient group (*n* = 10). Probiotic milk patient group (*n* = 12). (a) L-lactate; (b) D-lactate. Boxes represent 25th to 75th percentile of data and whiskers represent data spread. Asterisk (*) indicate that post treatment value is significantly different from baseline, *P *≤ 0.05. Please note the different scales in (a) and (b).

To further verify the higher lactate levels in the post treatment samples, a standard enzymatic analysis of the lactate concentration was performed (Figure 5). The lactate analysis determines the D- and L-isomer concentrations separately [27,28]. We found increased levels of L-lactate in post treatment samples of both treatment groups. No increase in D-lactate levels was found. Lactic acid bacteria can produce both lactate isomers and the body produces L-lactate. Based on our results, we can, therefore, only verify the increase in L-lactate but not conclude where the increase in L-lactate stems from. 

We tested for a probiotic effect by performing PCA and OPLS-DA on the difference between the post- and pre-treatment spectra (to remove baseline individual variation). This analysis did not reveal any difference in the response for the probiotic and GDL milk products. Nor were we able to see effects of gender, BMI, or a correlation with symptom relief [10] at this level.

## 4. Discussion

By employing ^1^H NMR spectroscopy in combination with multivariate data analysis, we revealed that consumption of 0.4 L of probiotic acidified milk product a day for eight weeks led to a change in the overall blood serum metabolite composition in IBS patients that was not different from that of non‑probiotic acidified milk. Since the experiment was designed to detect effects of probiotics in the context of an acidified milk product, and not the effect of the acidified milk itself, we cannot rule out effects from other factors. However, the clear and identical response in both treatment groups suggests a response to the delivering vector. We identified elevated levels of blood serum (L-) lactate and 3‑hydroxybutyrate after both treatments. 

The IBS patients enrolled in the present study consumed no probiotic dairy products during the two‑week wash-out period before entering the diet intervention period. Thus, the baseline samples correspond to a diet without any probiotic dairy products included. Under normal conditions, blood serum lactate levels are kept at a constant flux level in a dynamic process where cells release and take up lactate at a rate that depends on the metabolic rate of resting or exercising muscles [29,30,31,32].

Lactate ingested by consumption of the prescribed diet would thus be quickly removed after a meal [33,34]. The meal consumed one hour before both the baseline and the post treatment sample collection was devoid of dairy products. The observed effect was therefore independent of the meal consumed before sample collection. 

We identified the rise in lactate as a rise in the level of L-lactate. The elevated levels of 3‑hydroxybutyrate observed in both the GDL and the probiotic milk group can be correlated to the elevated L-lactate levels (*R* = 0.76 and *R* = 0.71 based on peak intensity, for the GDL treatment, and probiotic treatment groups, respectively), as conversion of lactate to glucose in the liver removes oxaloacetate from the tricarboxylic acid (TCA) cycle, which thereby reduces the utilization of acetyl‑CoA. Excess acetyl CoA will then be converted into ketone bodies such as 3‑hydroxybutyrate [35,36,37]. 

During the intervention period, symptom relief scores were recorded for all patients showing that a subgroup of the patients receiving the probiotic milk product experienced a faster relief in symptoms than patients receiving the GDL milk product. As the intervention progressed, the difference in symptom relief was balanced, and after eight weeks no discrimination between the two treatment types was found [10]. In this perspective, it is very interesting to see that we could not discriminate between the effects of added probiotics and GDL after eight weeks intervention. Symptom scores are subjective and based on the patient’s own evaluation, and high degrees of placebo response have been reported in diet intervention studies concerning bowel disorders [11,12,13]. Our results might suggest that the delivering matrix, in this case acidified milk, can have an influence on symptom relief in these patients. Although symptom relief only was observed for a subgroup of responders, the effect of the treatments at blood serum metabolite level was observed for the total patients in both treatment groups, and no correlations between symptom relief and metabolite response was observed. We can conclude that both responders and non-responders on both diets have similar serum levels of lactate and 3‑hydroxybutyrate after the intervention period, when the same level of symptom relief is observed for both diets. We were not able to find any correlation between the elevated levels of lactate and 3‑hydroxybutyrate and the symptom relief score, Thus, we cannot at this point state whether the serum metabolite response is directly related to the relief-causing mechanism or not. For this, further studies with samples taken throughout the intervention period are needed.

Both patients that received the GDL acidified milk product and patients that received the probiotic milk product had higher levels of blood serum lactate. Thus, the increase in lactate was independent of the presence of probiotics in the milk product. Reports have shown that a subgroup of IBS patients has an increased intestinal permeability [38,39,40], and paracellular permeability has also been linked to IBS [41,42]. Gut permeability caused by the syndrome could affect the lactate absorption. An increased production of lactate as a consequence of increased growth of intestinal lactic acid bacteria would lead to a higher serum level of lactate. Larger amounts of lactic acid-producing bacteria in the intestine, and hence more blood serum lactate, could be a result of either the large amounts of lactic acid bacteria consumed by the patients in the probiotic milk group, or of a favorable environment for native lactic acid-producing bacteria created by the intake of acidified milk products. Dietary records showed that patients clearly had a lower energy intake at the end of the trial period compared to before baseline samples were collected. However, patients consuming the GDL acidified product gained weight during the trial, while patients consuming the probiotic milk product neither gained nor lost weight during the trial period (Table 1). We did not observe any changes in lipid metabolite profiles indicating a response caused by the GDL treatment. Furthermore, we found the changes in metabolite profile after an eight week intervention period were identical for the two treatment types. We therefore propose that the observed metabolite changes were independent of the lower energy intake. 

## 5. Conclusions

Although no serum metabolite response to the probiotic diet intervention was found and the study did not contain any control for the effect of acidified milk, our results suggest that the delivering vector has an effect in itself. This means that it is very important to consider the delivering vector, when conducting probiotic diet intervention studies. The study revealed that the consumption of an acidified milk product gave rise to elevated levels of serum lactate and of 3-hydroxybutyrate, which we suggest to be a product of the high lactate condition observed after the intervention period. A lacking discrimination between the effects of the two diets is in good agreement with the questionnaire-based health-status results, though no correlations between symptoms relief and serum metabolite response was found.

## Declare

S.M.M. Pedersen and H.C. Bertram have a grant from The Danish Dairy Research Foundation. U. Svensson is Innovation Manager Bio Science at Arla Foods amba. H.J. Andersen is Head of Corporate Research at Arla Foods amba. J. Olsson, M. Simrén, L. Öhman and S.M.M Pedersen have received funding from Arla Foods amba. N.C. Nielsen and A. Malmendal have no competing interests.

## Supplementary Files

Supplementary File 1: Supplementary Material.pdf(PDF, 111KB)
