# Supplementary material for: The Serum Metabolite Response to Diet Intervention with Probiotic Acidified Milk in Irritable Bowel Syndrome Patients Is Indistinguishable from that of Non-Probiotic Acidified Milk by 1H NMR-Based Metabonomic Analysis
Source: Nutrients. 2010 Nov 23;2(11):1141–55. doi: 10.3390/nu2111141 (PMC3257620; doi:10.3390/nu2111141)

Supplemental material

Supplementary Table 1: Participant baseline characteristics (age, BMI, gender, time with and type of IBS) by treatment group. Supplementary Table 2: Composition of the acidified milk products. Supplementary Figure 1: PCA scoreplots of baseline and post treatment samples from both GDL treamtnet group. Supplementary Figure 2: PCA scoreplots of baseline and post treatment samples from probiotic milk treatment group. Supplementary Figure 3: OPLS-DA scoreplots of GDL and probiotic milk treatment groups.

Supplemental material – Pedersen *et al.* - The Serum Metabolite Response to Diet Intervention with Probiotic Acidified Milk in Irritable Bowel Syndrome Patients is indistinguishable from that of Non-Probiotic Acidified Milk by ^{1}H NMR-Based Metabonomic Analysis.

Supplemental Table 1. Participant characteristics by treatment group [a].

**Participant characteristic**	**GDL** [b]		**Probiotic** [c]	
Age, *y*	45.70	± 2.97	40.70	± 2.91
BMI, *kg/m²*	24.02	± 0.62	24.13	± 0.63
Gender, *%*				
Female	73		69	
Male	27		31	
Type of IBS, *%*				
Diarrhoea	48		40	
Constipation	28		20	
Constipation/Diarrhea	21		33	
No constipation or diarrhea [d]	3		7	
Time with IBS, *%*				
Less than 5 y	10		17	
More than 5 y	90		83	

[a] Age and BMI are given as means ± SEM. Gender, type of IBS and time with IBS are given as percentage of group. *P*(age) > 0.05 and *P*(BMI) > 0.05; [b] $n = 30$; [c] $n = 31$; [d] Constipation or diarrhea for this subgroup of patients was not recorded during the study period.

Supplemental Table 2. Composition of probiotic milk product and GDL milk product.

**Product**	**Probiotics-containing milk product**	**Acidified milk product**
Proximate composition (*g/kg*)		
Protein	40	40
Fat	15	15
Carbohydrate	40	65 [a]
Lactose	35	45
Ingredients	Milk	Milk
Acidifiers [b]	*Lactobacillus delbruckeii ssps. bulgaricus*<br>*Streptococcus thermophilus*	*D*-(+)-gluconic acid δ-lactone
Probiotics [c]	*Lactobacillus paracasei* F19<br>*Lactobacillus acidophilus* LA-5<br>*Bifidobacterium lactis* BB-12	

[a] Acidifier D-(+)-gluconic acid δ-lactone incorporated in the noted value; [b] Final concentration of biological fermenters in the product: $10^7 – 10^9$ CFU/g; [c] Final concentration of each probiotic strain in the product: $5x10^7$CFU/g.

Supplemental Figure 1. PCA scoreplot of blood serum baseline samples collected from the GDL and the probiotic milk product treated patient groups before the intervention period. *(A)* GDL milk treated patient group (*n* = 29). $R^2X(1)^a = 0.589$, $R^2X(2) = 0.149$ and $Q^2(\text{cum}) = 0.819$. *(B)* Probiotic milk treated patient group (*n* = 30). $R^2X(1) = 0.611$, $R^2X(2) = 0.197$ and $Q^2(\text{cum}) = 0.928$.

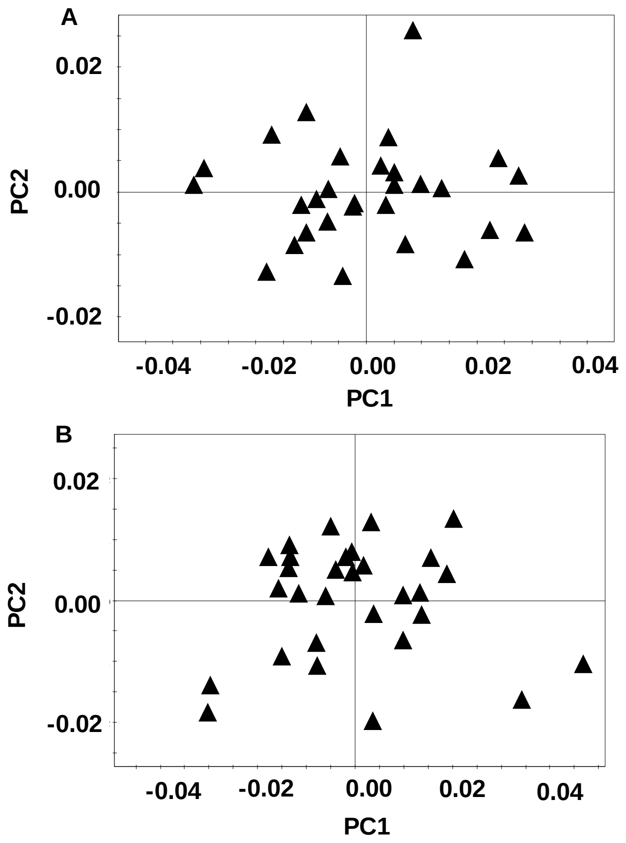

Supplemental Figure 2. PCA scoreplot of blood serum post treatment samples collected from the GDL and the probiotic milk product treated patient groups after an 8 wk intervention period. *(A)* GDL milk product treated patients (n = 29). $R^2X(1) = 0.592$, $R^2X(2) = 0.183$ and $Q^2(\text{cum}) = 0.923$. *(B)* Probiotic milk treated patient group (n = 30). $R^2X(1) = 0.638$, $R^2X(2) = 0.171$ and $Q^2(\text{cum}) = 0.925$.

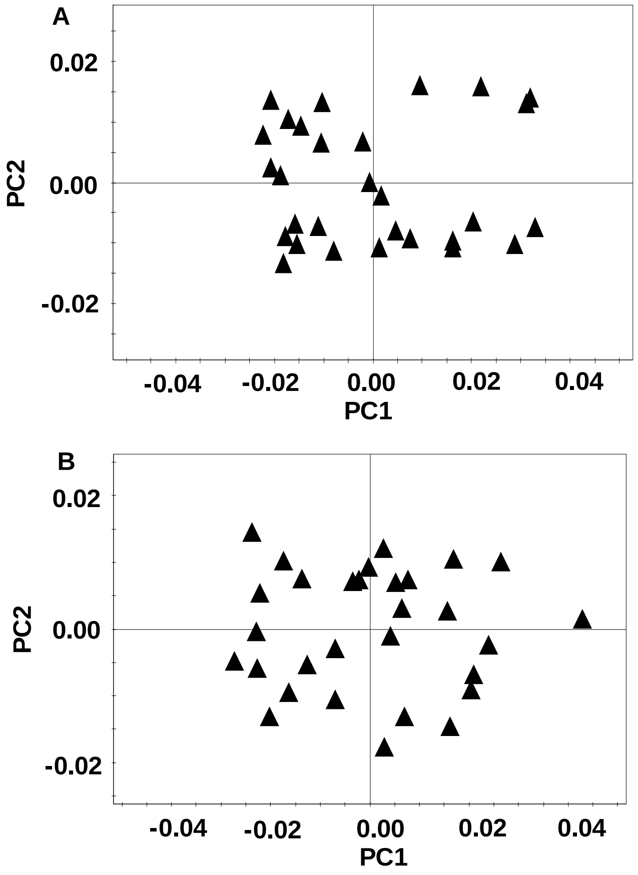

Supplemental Figure 3. OPLS-DA score plots of GDL and probiotic milk product treated patient blood serum samples. *(A)* OPLS-DA scoreplot of GDL milk patient group baseline and post treatment samples (n = 58). R^2X(cum) = 0.821 and Q^2(cum) = 0.841. *(B)* OPLS-DA scoreplot of probiotic milk patient group baseline and post treatment samples (n = 60). R^2X(cum) = 0.833 and Q^2(cum) = 0.848.

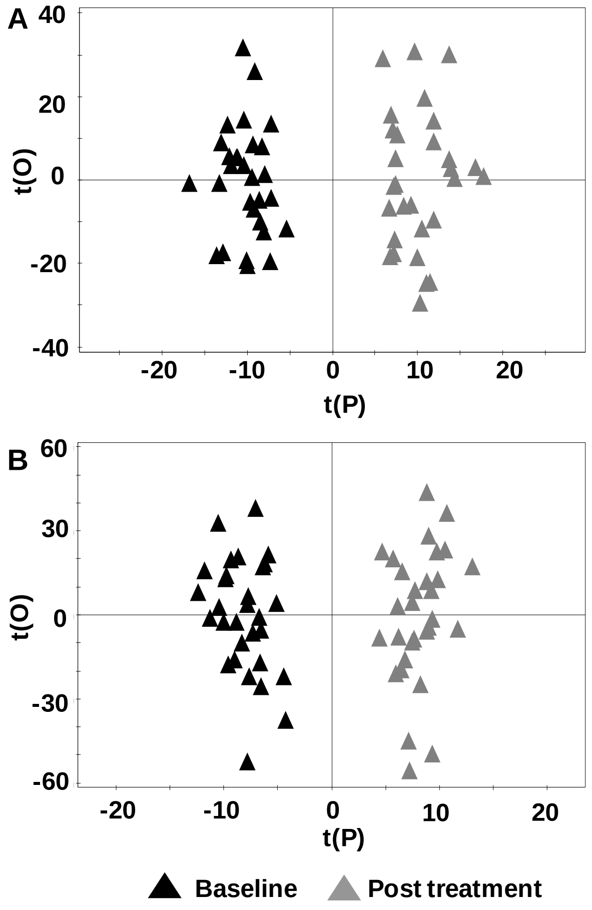

Supplement: Supplementary File 1: — Supplementary Material.pdf(PDF, 111KB) [file nutrients-02-01141-s001.pdf]
